# Optimization of an Anti-NMDA Receptor Autoantibody Diagnostic Bioassay

**DOI:** 10.3389/fneur.2018.00661

**Published:** 2018-08-22

**Authors:** Nan-Chang Chiu, Yi-Jie Lin, Ruu-Fen Tzang, Ying-Syuan Li, Hui-Ju Lin, Subir Das, Caleb G. Chen, Chiao-Chicy Chen, Kate Hsu

**Affiliations:** ^1^Division of Pediatric Neurology, Department of Pediatrics, MacKay Children's Hospital, Taipei, Taiwan; ^2^MacKay Junior College of Medicine, Nursing and Management, New Taipei City, Taiwan; ^3^MacKay Medical College, New Taipei City, Taiwan; ^4^Department of Psychiatry, MacKay Memorial Hospital, Taipei, Taiwan; ^5^MacKay Memorial Hospital Transfusion Medicine & Immunogenetics Laboratories, Tamsui, Taiwan; ^6^Institute of Biophotonics, National Yang-Ming University, Taipei, Taiwan; ^7^Department of Internal Medicine, MacKay Memorial Hospital, Taipei, Taiwan

**Keywords:** Anti-N-methyl-D-aspartate receptor (anti-NMDAR) encephalitis, autoantibody (autoAb), autoimmune encephalitis, schizophrenia, diagnostic test, fluorescein isothiocyanate (FITC), Alexa Fluor 488, antigen (Ag)

## Abstract

Anti-N-methyl-D-aspartate receptor (anti-NMDAR) encephalitis is one of the most frequently encountered autoimmune encephalitis. The pathogenesis of both anti-NMDAR encephalitis and schizophrenia involve down-regulation of NMDA receptors. Whether autoantibody-mediated destruction of neuronal NMDA receptors is associated with schizophrenia or first-episode psychosis (FEP) remains unclear, as the current findings from different groups are inconsistent. The main culprits are likely due to heterogeneity of autoantibodies (autoAbs) in a patient's blood or cerebrospinal fluid (CSF), as well as due to limitation of the current detection methods for anti-NMDAR autoAbs. Here, we optimized the current diagnostic method based on the only commercially-available anti-NMDAR test kit. We first increased detection sensitivity by replacing reporter fluorophore fluorescein isothiocyanate (FITC) in the kit with Alexa Fluor 488, which is superior in resisting photobleaching. We also found that using an advanced imaging system could increase the detection limit, compared to using a simple fluorescence microscope. To improve test accuracy, we implemented secondary labeling with a well-characterized mouse anti-NR1 monoclonal antibody (mAb) after immunostaining with a patient's sample. The degree of colocalization between mouse and human antisera in NMDAR-expressing cells served to validate test results to be truly anti-NMDAR positive or false-positive. We also incorporated DNA-specific DAPI to simultaneously differentiate autoAbs targeting the plasma membrane from those targeting cell nuclei or perinuclear compartments. All the technical implementation could be integrated in a general hospital laboratory setting, without the need of specialized expertise or equipment. By sharing our experience, we hope this may help improve sensitivity and accuracy of the mainstream method for anti-NMDAR detection.

## Introduction

Diagnosis of anti-NMDAR autoimmune encephalitis requires identification of pathogenic anti-NMDAR autoAbs in a clinical sample (1). Because anti-NMDAR autoAbs could target neuronal receptor and impair glutamatergic transmission, psychotic and cognitive disturbing symptoms are prominent in anti-NMDAR encephalitis (2–4, 5). Not surprisingly, early presentation of anti-NMDAR encephalitis shares symptoms of schizophrenia. For neuropsychiatrists, it has been an intriguing research topic to determine whether autoantibodies against NMDA receptor might contribute to the pathogenesis of a subset of schizophrenia through autoimmune-mediated neuroinflammation.

In an early study of 571 patients diagnosed with anti-NMDAR autoAbs, 23 of them (4%) presented no neurological symptoms but isolated psychiatric episodes (6). As many patients with anti-NMDAR encephalitis are first seen by psychiatrists for their initial prominent psychiatric symptoms, these 4% of the anti-NMDAR-positive patients with only psychiatric symptoms conceivably might be diagnosed as psychosis or even schizophrenia in psychiatric clinics. In our hospital psychiatric clinics in Taiwan, we have identified anti-NMDAR autoAbs in first-visited patients who showed abrupt and atypical psychosis with autonomic disturbance. After the correct diagnosis, their psychiatric symptoms were eventually cured by immunosuppressive treatments, emphasizing the extreme importance of correctly sorting out these patients (7, 8).

Similar results were found by research teams in U.K. and Japan (9–11). In U.K., Zandi and colleague reported the presence of serum anti-NMDAR autoantibodies in 6.5% of the patients with schizophrenia (9); Lennox et al. reported anti-NMDAR IgG in 3% of 228 patients with FEP and not in the blood samples of 105 healthy controls (11). In Japan, Tsutsui and colleague found anti-NMDAR autoAbs in the sera of four out of 51 patients with schizophrenia and schizoaffective disorder (7.8%) (10). However, there are contradicting findings from groups in Germany (0.7% anti-NMDAR IgG in 1081 schizophrenic patients and 0.4% in 1272 healthy subjects) (12), in another Taiwan hospital (0% in 78 patients with first-episode schizophrenia and 0% in 234 patients with chronic schizophrenia) (13), and in Turkey (0% in 49 schizophrenic patients and 0% in 48 healthy subjects) (14). Thus, whether anti-NMDAR autoantibodies could be associated with pure psychiatric illness (e.g., schizophrenia) remains an open question.

Though autoantibodies present in autoimmune patients are intrinsically complex and heterogeneous with a diverse range of specificities and affinities to autoantigens, the root of these controversies likely also involves the various detection approaches that different research groups took. The current detection methodology for anti-NMDAR autoAbs, whether developed commercially or in-house in individual academic labs, all utilizes NR1/NR2-expressing cultured cells for immunofluorescence labeling (9–11, 15). In this approach, negative controls are untransfected or untransduced cultured cells; tester cells are NR1/NR2-expressing cultured cells. A test result is considered positive if the blood or CSF sample from a patient shows reactivity to heterologously-expressed NMDA receptor, and not to negative-control cells.

Research groups that incorporate their in-house immunostaining protocols generally reported higher occurrence rates of anti-NMDAR autoAbs in patients with schizophrenia or psychosis, and absence or lower frequencies of the antibodies in healthy controls (9, 11, 15–17). Because the in-house protocols generally use live NR1/NR2b-expressing cultured cells, they provide a broader and more realistic range of antigenic sites than chemically-fixed cells from a commercial kit. However, heterologous expression of NMDA receptor in cultured cells requires ketamine, which is inaccessible to most laboratories including ours. So we also used the conventional kit for anti-NMDAR tests.

As suggested in recent Commentaries and Replies to journal articles, the different results from different groups might also be related to the imperfect performance of the commercial kit that many of them relied on (4, 9, 17–22). Based on our experience with the commercial reagents, there were definitely rooms for improvement. In our early trials with these reagents, we noticed that the fluorescent signals that reported antibody-antigen (Ab-Ag) interaction quenched quickly under a conventional fluorescence microscope. We initially often had to repeat a test several times, especially for clinical samples that were eventually determined to have low titers of anti-NMDAR autoAbs. This report described our approaches to increase sensitivity and accuracy of anti-NMDAR detection based on the conventional approach.

## Materials and methods

### Ethics statement

The study was carried out in accordance with the principles of the Declaration of Helsinki, and was approved by the Institutional Review Board (IRB) of Taiwan Mackay Memorial Hospital (MMH)(MMH-IRB registration numbers: 14MMHIS068 & 14MMHIS282). Written informed consent was obtained from all participating subjects.

### Optimization for detection of anti-NMDAR autoAbs

Optimization was based on the recommended protocol of the anti-Glutamate Receptor (type NMDA) IIFT kit (EUROIMMUN, Lubeck, Germany). Similar to the Manufacturer's Instruction, 30 μl of a clinical sample (either undiluted or 1:2 diluted CSF, or 10-fold diluted blood serum or plasma) was incubated with a pair of Tester and Negative-Control BIOCHIPs for 1 hour at room temperature, followed by two washes with PBS-Tween 20 (all provided by the kit) for 5 min. Tester and Negative-Control BIOCHIPs are mini chips coated with fixed, NMDAR-expressing cultured cells and unexpressed cells, respectively. These paired chips are embedded on a microscope slide. In the protocol provided by the kit, ab-ag interaction was probed by secondary labeling with 25 μl of FITC-conjugated anti-human antisera (included in the kit) for 30 min at room temperature, followed by washes.

To increase detection sensitivity, we substituted secondary FITC-conjugated anti-human antisera (provided in the kit) with Alexa fluor 488-conjugated anti-human immunoglobin (1:100 dilution; Jackson ImmunoResearch Laboratories, West Grove, PA, USA). Detailed optimization for the concentration of detection probe Alexa Fluor 488, incubation time, and dilution of clinical samples was provided in the online Supplemental Figures. Figure S1 showed the optimal dilution of Alexa Fluor 488-conjugated anti-human immunoglobin to be 1:100. Figure S2 showed the optimal length of time for incubating a clinical sample with BIOCHIPs to be 1 hour. Figure S3 showed how an autoAb titer is determined using a blood plasma sample. For titer determination, a plasma/serum sample is generally diluted at 1:10, 1:32, 1:100, 1:320, and up to 1:640. An autoAb titer is the highest possible dilution that still allows for visualization of the fluorescence signals from the antibody-antigen interaction. Figure S4 showed the ideal dilution of a CSF test to be 1:2 or no dilution (the latter identical to the manufacturer's suggestion) using our immunostaining protocol.

### Double immunolabeling with mouse anti-NR1 mAb

For clinical samples with ambiguous test results using the single-labeling method (described above), the samples could be re-tested or tried with the double-labeling method to verify the accuracy of single-staining results. For double labeling, a specific mouse anti-NR1 mAb is incorporated to mark subcellular locations that express NMDA receptor. The rationale is that if a patient's sample does not react to the same subcellular regions as the mouse mAb, then this patient does not have anti-NR1 autoAbs. The first part of the double-labeling protocol was identical to single labeling described above. Briefly, 30 μl of a clinical sample was incubated with a pair of Negative-Control and Tester BIOCHIPs for 1 hour, followed by washes. An optional fixative step with 0.01% glutaraldehyde for 30 seconds could be employed prior to second labeling with mouse anti-NR1 mAb clone 54.1 (1:2000 dilution; Merck-Millipore, Temecula, CA, USA). The second staining with this specific mouse mAb lasted for 1 hour, followed by washes. Though human and mouse antisera were incubated with BIOCHIPs sequentially, their individual labeling with the detection fluorophores were administered simultaneously. Specifically, after sequential probing with human and mouse antisera, the BIOCHIPs were then incubated in 25 μl of a mixture of Alexa fluor 488-conjugated anti-human and Alexa fluor 568-conjugated anti-mouse immunoglobin for 30 min (both at 1:1000 dilution; both from Jackson ImmunoResearch Laboratories), followed by washes.

For labeling of cell nuclei, instead of using glycerol from the commercial kit, immunostained BIOCHIPs were sealed with a glycerol-based mountant containing DAPI (Thermo Fisher Scientific, Waltham, MA, USA).

### Fluorescence imaging and calculation

The images were taken from (1) an inverted fluorescence phase contrast microscope (OLYMPUS IX71) coupled with the SPOT RT3 microscope digital camera and imaging processing system (Diagnostic Instruments Inc., Sterling Heights, MI, USA); (2) the TissueFAXS Cell Analysis System (TissueGnostics GmbH, Vienna, Austria). In double-labeling experiments, the degree of colocalization (R_coloc_) between green and red fluorescence was calculated by the Colocalization Threshold plugin (ImageJ). R_coloc_ is the Pearson's correlation coefficient for images above thresholds: R_coloc_~1 refers to a perfectly positive correlation; R_coloc_~0 refers to the complete absence of a correlation (23). For verification of image-based test results, the batch number of each BIOCHIP-embedded slide was recorded. Immunostained BIOCHIPs were examined independently by at least two lab specialists under a fluorescence microscope. The criteria and workflow for the optimized anti-NMDAR autoAb diagnostic test are outlined at the end of this paper.

## Results

### Improvement of anti-NMDAR detection sensitivity

**Figure 1:**
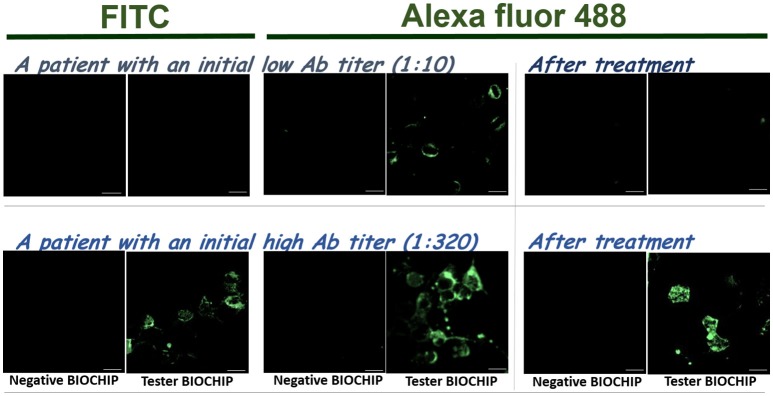
Replacement of secondary anti-human antisera-conjugated fluorophore FITC (left) with Alexa Fluor 488 (right) improved the sensitivity of anti-NMDAR autoAb detection based on EUROIMMUN's anti-Glutamate Receptor IIFT. The experimental procedure mostly followed the recommendation from the manufacturer. **Top:** Comparison using a clinical sample with a low content of anti-NMDAR autoAbs (titer 1:10). **Bottom:** Comparison using a sample with a high titer of anti-NMDAR antisera (titer 1:320). The right pairs are from the same patients after effective immunosuppressive treatments. Scale bars, 20 μm.

To improve detection sensitivity, we compared the reporter fluorescence probe provided in the kit, FITC, with functionally-equivalent Alexa fluor 488. Though both fluorophores are nearly identical in spectral properties (excitation max 490 nm / emission max 525 nm) and quantum yields (~0.9), Alexa fluor 488 is significantly more photostable and less sensitive to environmental changes (e.g., pH), and has higher initial brightness (24, 25). In Figure 1, labeling with either FITC or Alexa fluor 488 gave strong signals for a sample with a high titer of anti-NMDAR autoAbs (Figure 1, bottom). However, for a sample with a low anti-NMDAR titer, labeling with Alexa fluor 488 showed distinctive differences between negative-control and NMDAR-positive BIOCHIPs, while that differences were much less distinguishable with FITC (Figure 1, top). After effective immunosuppressive treatments, both cases showed visibly reduced titers of anti-NMDAR autoAbs (Figure 1, right panels). For the patient with an initial low autoAb titer (1:10), his autoAbs after the treatments became almost undetectable even with more sensitive Alexa Fluor 488 (Figure 1, right panels).

Table 1 showed that Alexa Fluor 488 replacing FITC as the detection probe improved detection sensitivity, and generally allowed higher sample dilution or titers. Labeling with more sensitive Alexa fluor 488 also allows a broader range of autoAb detection, as reflected by the broader range of autoAb titers in the same patient samples that were also tested with FITC (Table 1). We also tested 26 stable psychiatric patients (23 schizophrenia and 3 bipolar disorder) from the hospital psychiatric day-care center, and found anti-NMDAR autoAb present in one out of the 26 patients (with a weak blood titer at 1:32). This patient suffered from chronic schizophrenia, and did not meet the criteria for possible autoimmune encephalitis (1). Thus, we were able to identify anti-NMDAR autoAb in ~3.8% psychiatric patients using the improved, kit-based method. Our positive rate for anti-NMDAR autoAb in psychiatric patients was similar to the rates reported by various groups using their more sensitive, in-house-developed methods described in the Introduction section (9, 10, 15–17). For comparison, our improved tests on 101 healthy control samples did not yield a positive result (Table 1).

**Table 1:** Comparison of the sensitivity of anti-NMDAR test between the two detection probes–FITC and Alexa Fluor 488.

Subject^*^	**Status of anti-NMDAR encephalopathy**	**Anti-NMDAR titer**^**#**^	
		**FITC**	**Alexa Fluor 488**
pt 1	Cured	1:10	1:32
pt 2	Cured	1:10	1:10
pt 3	Cured	1:10	1:32
pt 4	Recurrent	1:32	1:320
pt 5	Recurrent	1:32	1:100
pt 6	Cured	Indeterminate	1:10
Psychiatric day-care patients^**^(n = 26)	–	–	25 negative; 1 positive (blood titer 1:32)^##^
Healthy controls *(n = 101)*	–	–	All negative *(101/101)*

* *Patient (pt) subjects diagnosed of anti-NMDAR encephalitis fulfilled the diagnostic criteria listed in Graus et al. (1)*.

** *These were stable psychiatric patients attending programs at MMH Psychiatric Day-Care Centre: 23/26 schizophrenia; 3/26 bipolar disorder*.

# *The anti-NMDAR titer was determined by the highest possible dilution of a patient's plasma or serum sample which could still reveal fluorescence signals from anti-NMDAR autoAb labeling*.

## *The only blood anti-NMDAR-positive patient is a stable patient with schizophrenia, whose symptoms do not meet the criteria for possible autoimmune encephalitis (1)*.

**Figure 2:**
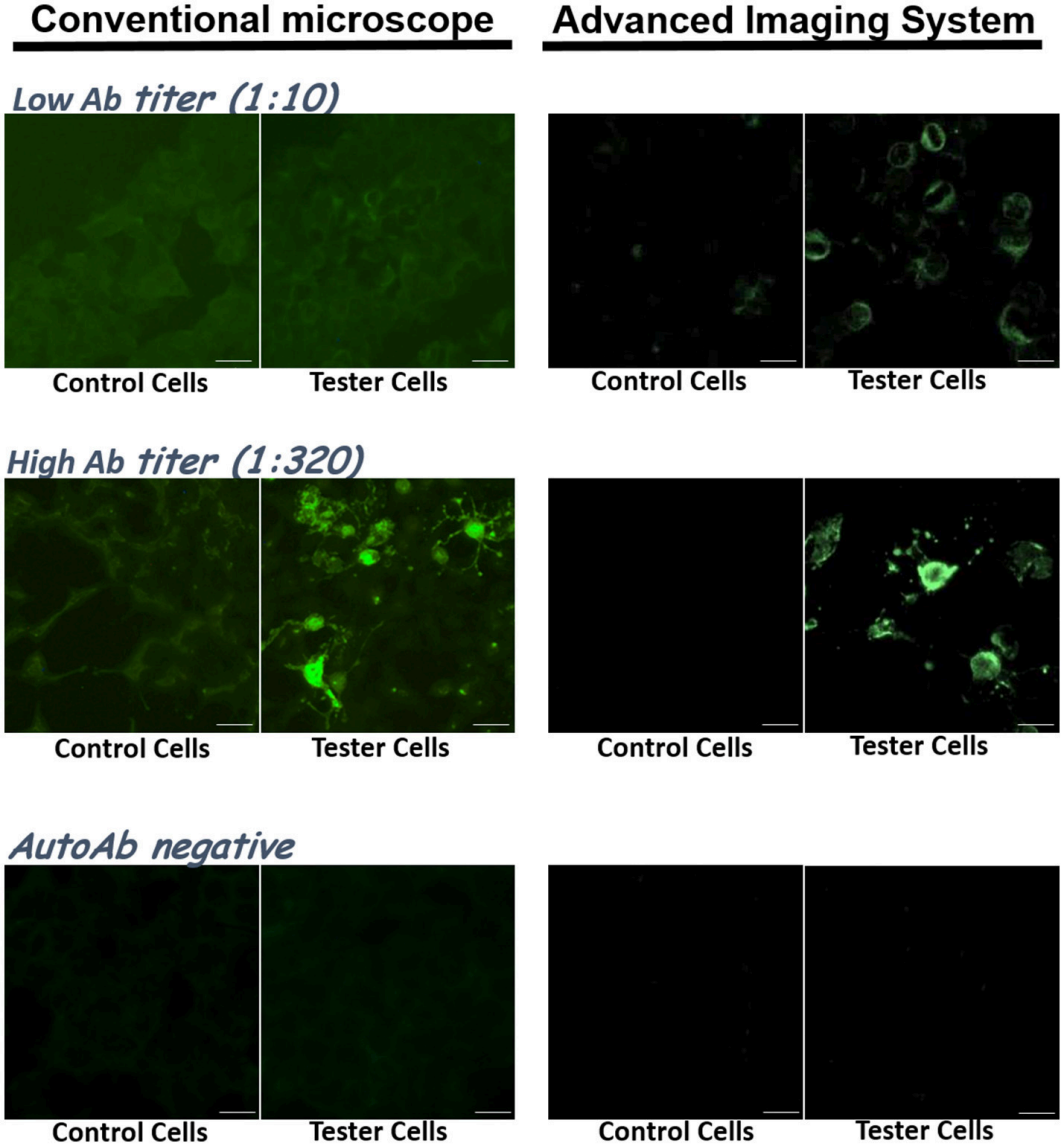
The choice of optic/imaging systems could affect resolution and sensitivity of anti-NMDAR detection. Clinical samples were tested with the IIFT kit, and their images taken by a conventional fluorescence microscope (left) and by an advanced imaging system from TissueGnostics GmbH (right) were compared. **Top:** images from a sample with a low anti-NMDAR titer (1:10). **Middle:** images from a sample with a high anti-NMDAR titer (1:320). **Bottom:** images from an anti-NMDAR-negative sample. Scale bars, 20 μm.

From our early trials using five different types of fluorescent microscopes/imaging systems in our department, we found that the choice of an imaging system affected detection sensitivity. Most of the imaging systems were able to resolve samples with high antibody titers (e.g., 1:100 or higher) (Figure 2, middle). A high-end optic/imaging system could further resolve relatively weak signals from samples with low Ab titers (Figure 2, top).

### Improvement of anti-NMDAR detection accuracy by co-labeling with a mouse anti-NR1 mAb

There was a need to improve the accuracy of anti-NMDAR detection. We occasionally encountered uncertain readouts that showed no staining in the negative-control cells but positive signals with unusual patterns in NMDAR-expressing cells. These unusual staining patterns could appear punctate, absent from the plasma membrane, or present in unexpected subcellular locations (e.g., cell nuclei). The percentage of fluorescence-labeled cells in the BIOCHIPs could also be used to roughly assess the accuracy of a test result. We estimated experimentally that not all but about 30–50% of the cells on the EUROIMMUN's Tester BIOCHIP express NMDA receptor. So experimenters should be alert when only sporadic cells or over 50% of the cells on a Tester BIOCHIP are fluorescently labeled.

**Figure 3:**
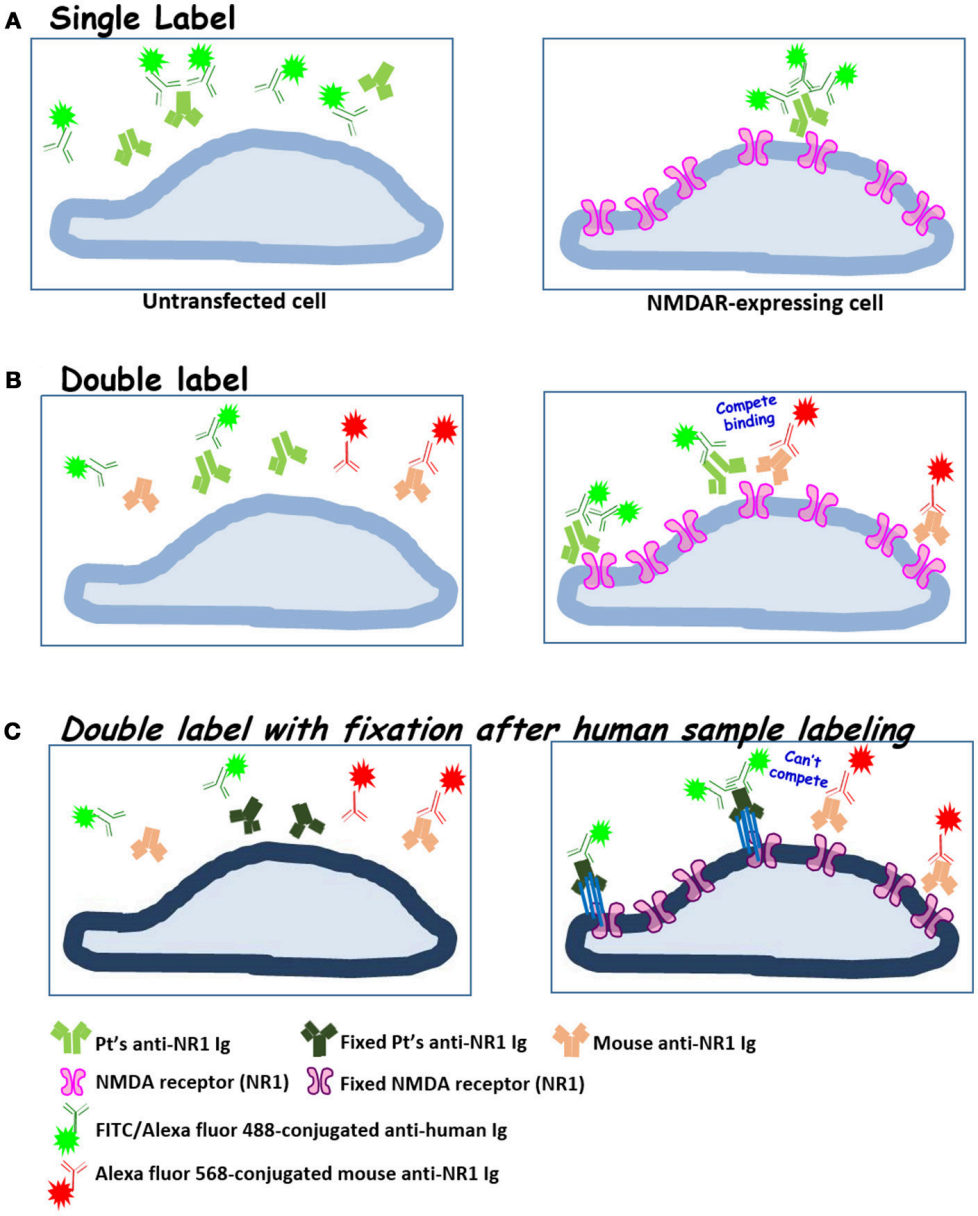
The diagrams illustrate our experimental approaches to validate anti-NMDAR test results by double labeling with a well-characterized mouse anti-NR1 mAb. **(A)** Single labeling with human blood or CSF samples (standard protocol); **(B)** sequential double labeling that starts with a human sample (green) and then a mouse anti-NMDAR mAb (orange-red); **(C)** incorporation of brief fixation (blue bars indicating chemical crosslinkers) after clinical sample labeling. Mouse anti-NR1 mAb thus can no longer compete with human antisera for binding to NMDA receptor, as in **(B)**. Fixed cell membrane and proteins were represented in darker hues.

**Figure 4:**
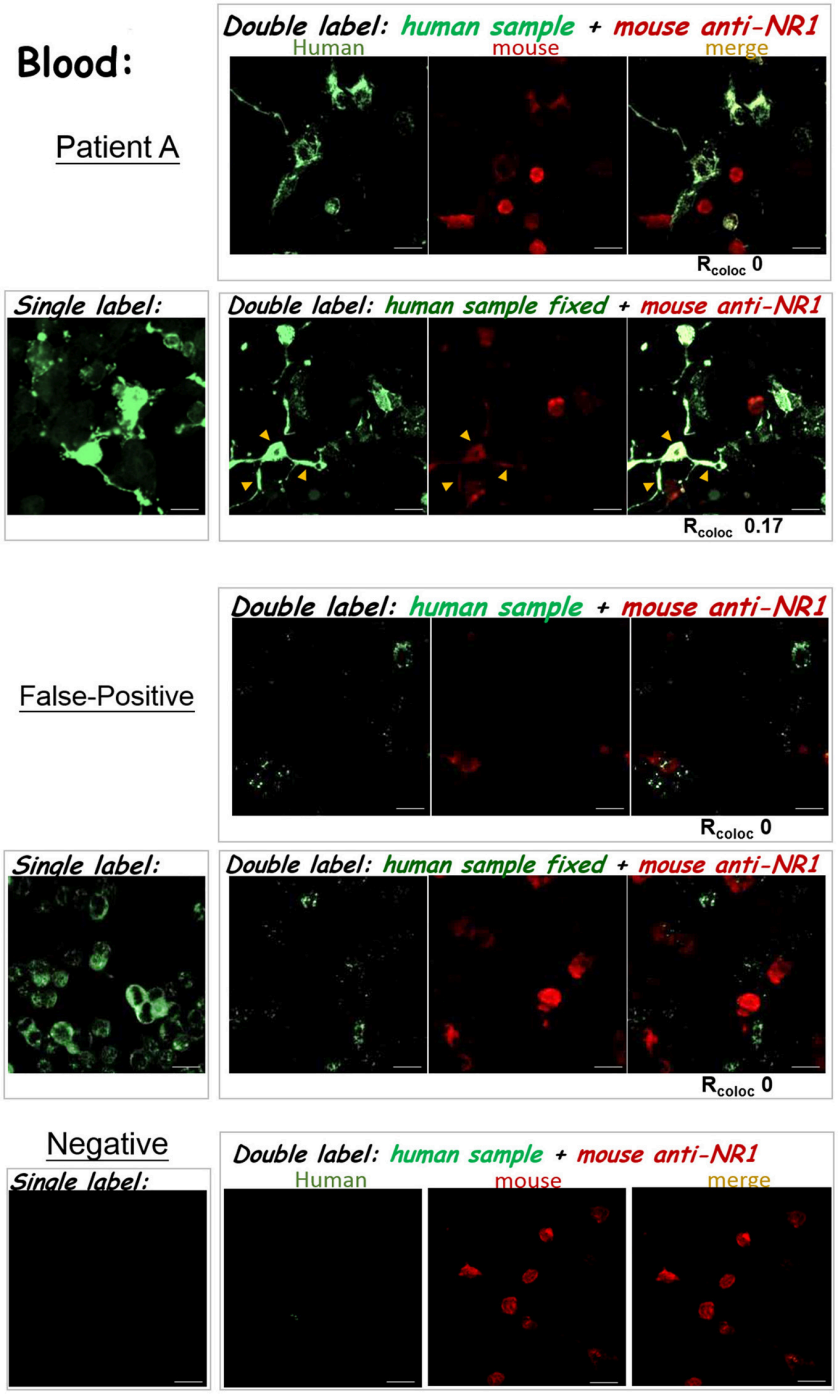
The results of anti-NMDAR autoAb tests were confirmed by double labeling. The three experimental protocols utilizing the IIFT kit (as illustrated in Figures 3A–C) were compared. The degree of green/red colocalization, represented by R_coloc_, was indicated beneath each dual-color merged image. **Top**—Patient A: The degrees of colocalization between a diluted plasma sample from patient A and the mouse anti-NR1 mAb improved after incorporating glutaraldehyde fixation following sample labeling (R_coloc_ ~0 → ~0.17). **Middle**—False-positive: A diluted blood sample showed positive signals by the standard single-labeling protocol (left images), and were later deemed “false-positive” by both double-labeling tests (right panels). This sample with “false-positive” results failed to colocalize with heterologously-expressed NMDAR by either tests illustrated in Figures 3B,C. **Bottom**—anti-NMDAR-negative: No green fluorescence was shown in the Tester BIOCHIPs by single or double labeling with an anti-NMDAR-negative plasma sample. Yellow arrowheads pointed to sites of colocalization of green and red fluorescence (overlay in yellow-orange color). Scale bars, 20 μm.

To improve the accuracy of anti-NMDAR detection, it is critical to verify whether ambiguously positive signals indeed result from binding to NMDA receptor, and not from interaction with cellular components other than NMDA receptor on a Tester BIOCHIP. We implemented second labeling with a well-characterized mouse anti-NR1 mAb clone 54.1 to specifically locate heterologously-expressed NMDA receptor (15), after labeling the BIOCHIP with a clinical sample. This mouse anti-NR1 was stained with red fluorescent Alexa Fluor 568, while bound human autoAbs were stained with green fluorescent Alexa fluor 488 (Figure 3). Noticeably, for some clinical samples, this mouse anti-NR1 might compete with a patient's autoAbs for binding to NMDA receptor, resulting in a low degree of colocalization between the mouse and the human antisera. To circumvent the issue, we added a brief fixative step following clinical sample labeling and before labeling with mouse anti-NR1. This fixative step could prevent mouse anti-NR1 from outcompeting a patient's autoAbs for binding to NMDA receptor (Figure 3), since mouse mAb clone 54.1 generally exhibits higher affinities to NMDA receptor than most human anti-NMDAR antisera. In some tests, indeed this additional fixative step increased % colocalization between mouse and human anti-NMDAR antisera [Figure 4—patient A: R_coloc_ ~0 (without fixation) vs. R_coloc_ ~0.17 (with fixation)].

By marking heterologously-expressed NMDA receptor with red fluorescence on a Tester BIOCHIP, this double-labeling approach allowed us to identify “false-positive” results. As demonstrated in the second example in Figure 4, this initial test result by single staining showed green fluorescent cells in the Tester BIOCHIP and no signals in the Negative-Control BIOCHIP. Our experimenter however noticed that almost all the cultured cells on the Tester BIOCHIP were green fluorescent, and decided to re-test this sample by double staining. Experimentation with either double-labeling approaches, with or without fixation following clinical sample labeling, failed to identify any colocalization between the human sample and the mouse mAb in NMDAR+ cells on the Tester BIOCHIP. This clinical sample was thus considered “false-positive,” since the green fluorescent signals shown on the Tester BIOCHIP did not result from binding to NMDA receptor.

**Figure 5:**
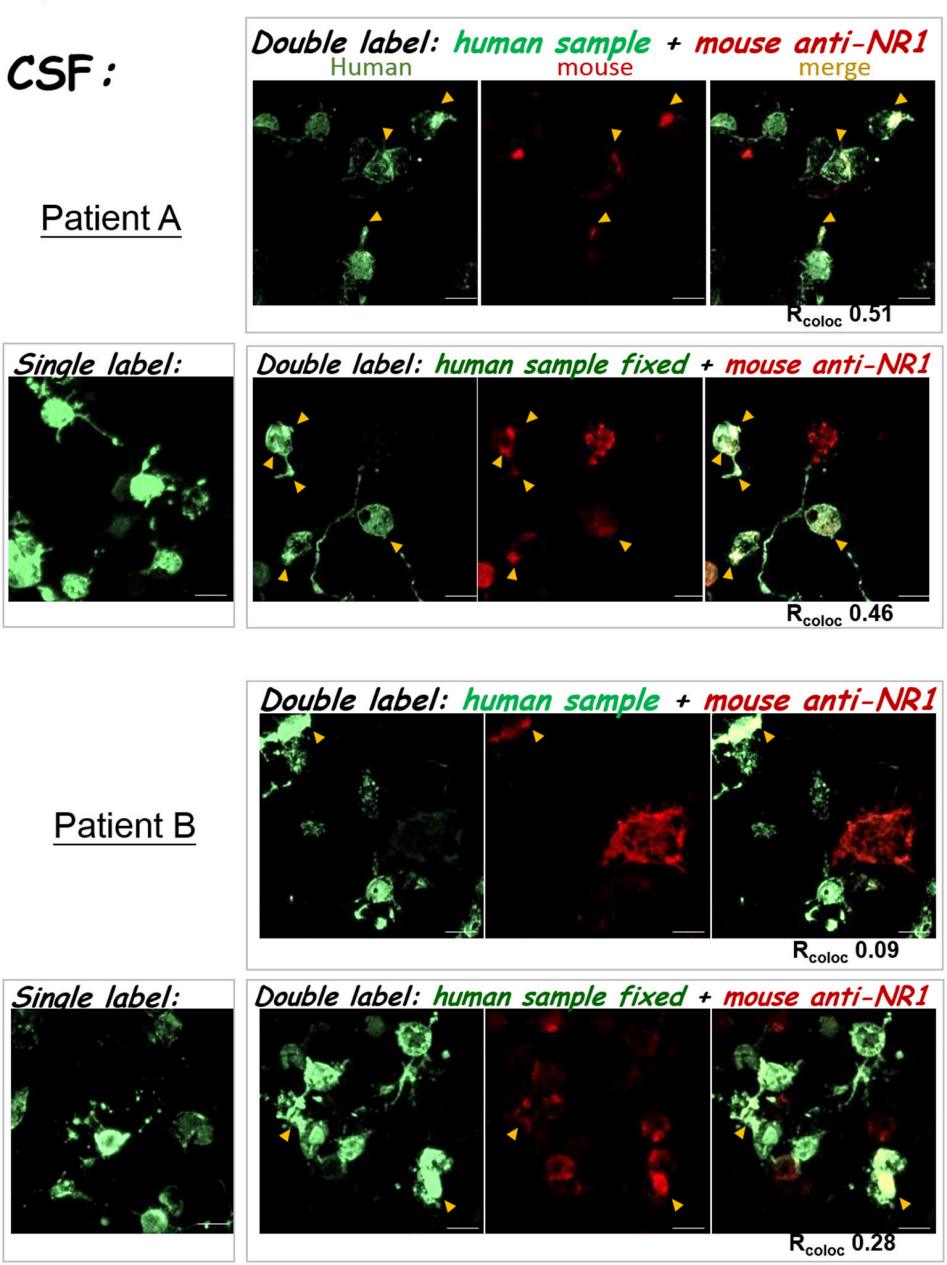
Double labeling with the mouse anti-NR1 mAb confirmed test results for clinical CSF samples. The left images were single-labeling results for the two CSF samples. The right images were from the two double-stain protocols (as in Figures 3B,C). **TOP:** CSF test results from Patient A. **BOTTOM:** CSF test results from patient B. Yellow arrowheads pointed to sites of colocalization of green and red fluorescence (overlay in yellow-orange color). Scale bars, 20 μm.

This double-labeling approach could also be used to verify CSF test results. As demonstrated in the second example in Figure 5, incorporation of a fixative step after staining with the patient's sample and before staining with the mouse anti-NR1 also increased % colocalization (Figure 5 bottom—patient B: R_coloc_ ~0.09 [without fixation] → R_coloc_ ~0.28 (with fixation)). Intriguingly, the CSF autoAbs of patient A exhibited similar degrees of colocalization with the mouse anti-NR1, regardless of whether there was a fixative step after initial sample labeling [Figure 5 top—patient A: R_coloc_ ~0.51 (without fixation) vs. 0.46 (with fixation)]. But for the blood sample of patient A, the degree of antibody colocalization was enhanced with fixation (Figure 4, top). These differences suggest that the avidities or the composition of anti-NMDAR autoAbs from the CSF and from the blood samples of patient A were different, because they were both compared experimentally to the same mouse anti-NR1 clone.

### Differentiation of anti-NMDAR from anti-nuclear autoAbs

**Figure 6:**
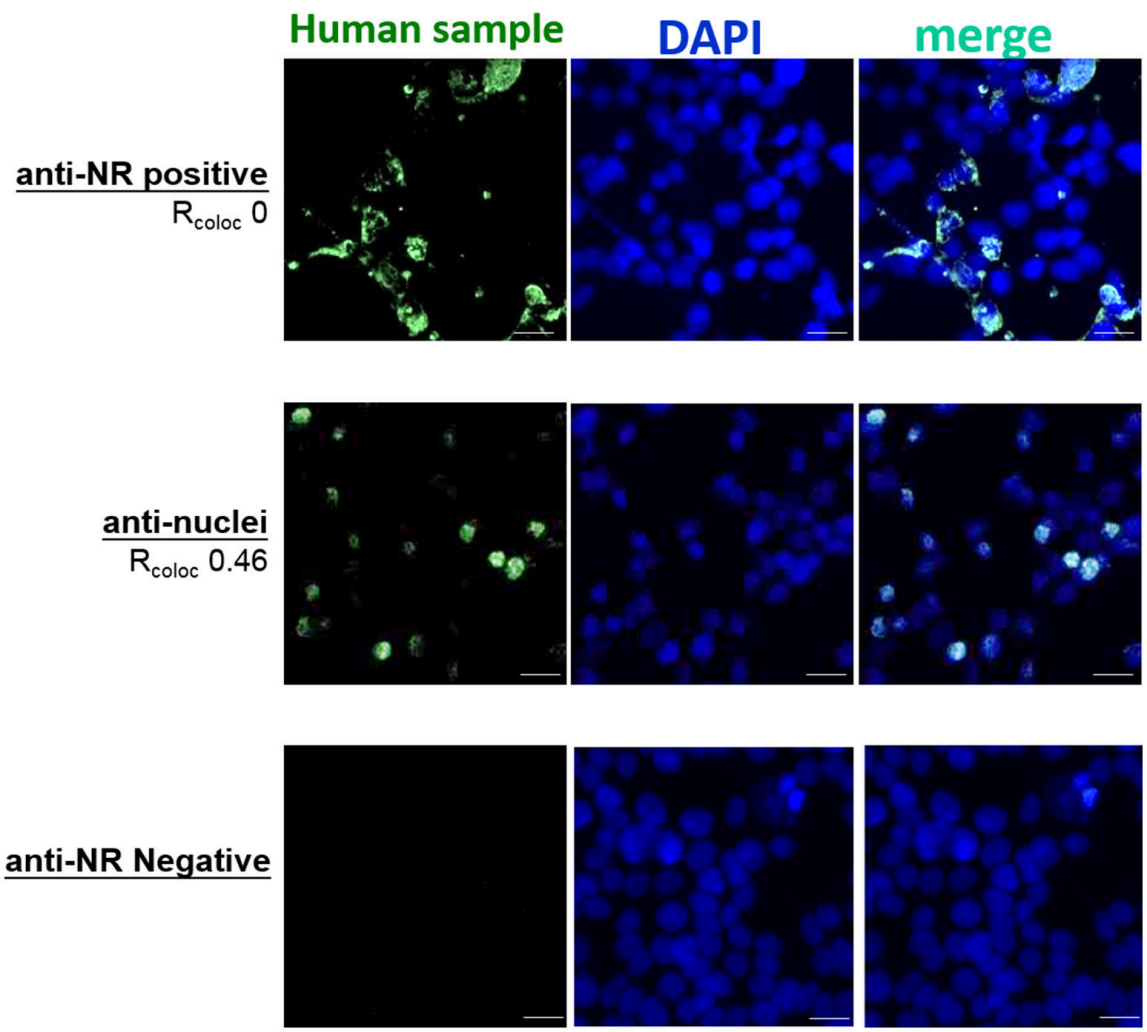
Inclusion of DAPI stain in anti-NMDAR tests helped differentiate immunostaining patterns by anti-NMDAR antibodies from that by nucleic acid-reactive substances. The degrees of colocalization between labeling of a clinical sample (green fluorescence) and labeling by DAPI (blue fluorescence) were expressed in R_coloc_. The images from an anti-NMDAR (NR)-positive clinical sample (top example: R_coloctoDAPI_ ~0) and a clinical sample primarily reacting to cell nuclei (middle example: R_coloctoDAPI_ ~0.46) were put together for comparison. The images from an anti-NMDAR-negative sample showed no green fluorescence (bottom example). Scale bars, 20 μm.

We also improved the accuracy of anti-NMDAR diagnostics by adding DAPI. This allowed experimenters to evaluate immunostaining patterns of clinical antisera, and to simultaneously identify all cell nuclei and estimate the percentage of positively-stained cells in a tester BIOCHIP. As NMDA receptors are expressed on the plasma membrane and the membranes of endoplasmic reticulum and the Golgi apparatus, the degree of colocalization between anti-NMDAR antisera and cell nuclei should be zero or extremely low (Figure 6 top: an NMDAR-positive sample with R_coloctoDAPI_ ~0).

By DAPI labeling, we had found an anti-NMDAR-positive sample that also showed substantial colocalization to cell nuclei in the Tester BIOCHIP (Figure 6 bottom: R_coloctoDAPI_ ~0.46), but not to cell nuclei in the Negative-Control BIOCHIP. Notably, heterologous expression of NMDA receptors requires NR2, which is also an autoimmune target in neuropsychiatric systemic lupus erythematosus (NPSLE) (26, 27). So we suggested further testing and evaluation for this patient. Thus, incorporation of DAPI stain in an anti-NMDAR test could potentially help differentiate anti-NMDAR encephalitis from other types of autoimmune insults in the CNS.

### A workflow for lab testing of anti-NMDAR autoAbs

**Figure 7:**
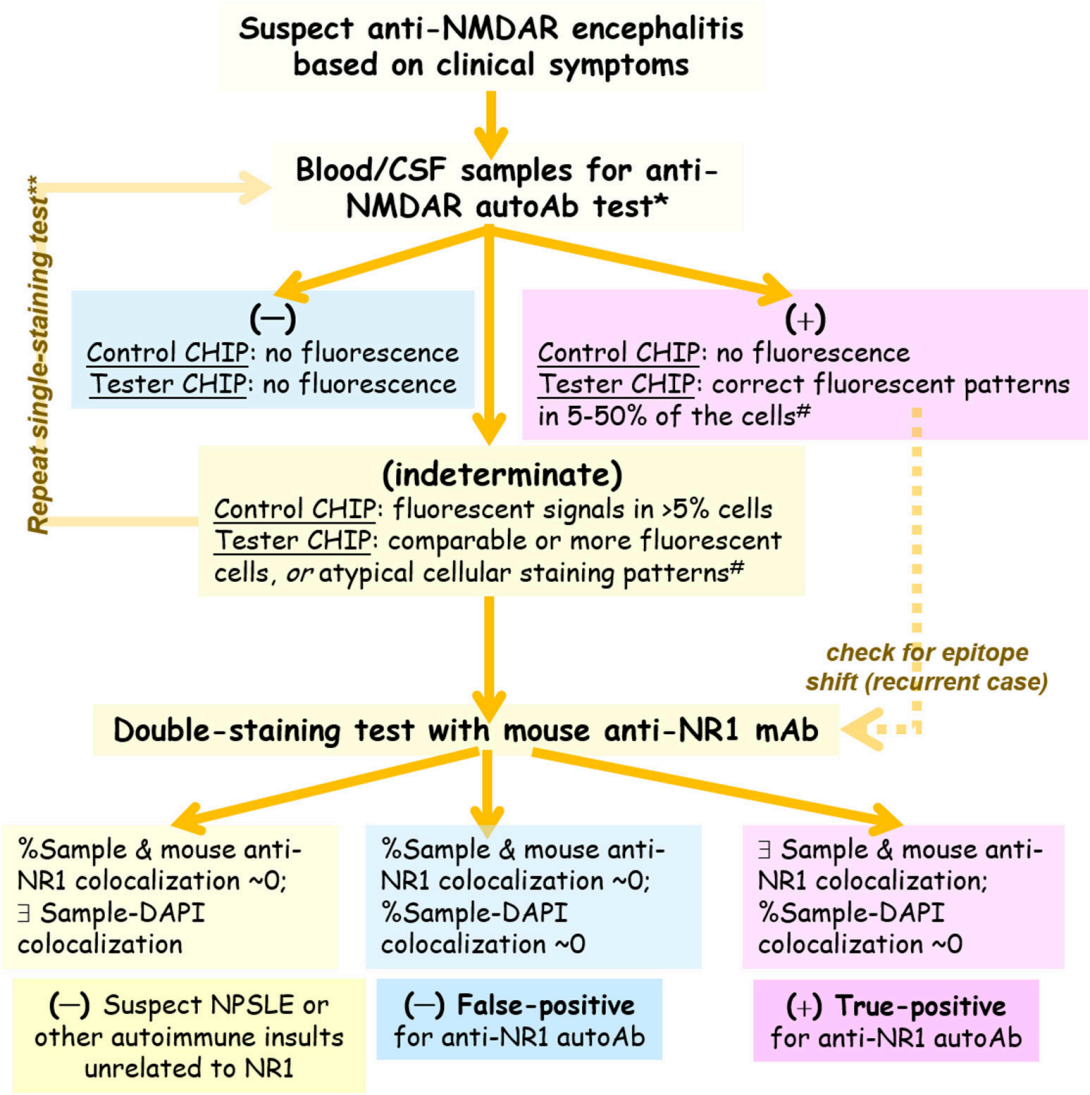
An optimized workflow for anti-NMDAR diagnostic bioassay. *The anti-NMDAR autoAb test utilizes reagents from EUROIMMUN's IIFT kit, with two changes: (1) the detection probe FITC provided by the kit is replaced by Alexa fluor 488 for higher sensitivity; (2) glycerol provided by the kit is replaced by a DAPI-containing mountant for marking cell nuclei. ^#^An atypical cellular staining pattern does not reflect the normal subcellular localization of membrane receptor such as NMDAR (e.g., fluorescent signals absent from the plasma membrane or present inside cell nuclei). **After reporting an indeterminate result to the physician in charge, he or she may request re-testing using the same sample or using a newly-withdrawn sample.

Table 2 summarizes anti-NMDAR tests performed with our optimized single-labeling protocol and some further validated with the double-labeling protocols. For convenience and consistency of lab testing, our workflow (Figure 7) utilized all the reagents from the anti-Glutamate Receptor IIFT kit but the secondary FITC probe and glycerol. We replaced FITC with superior fluorophore Alexa Fluor 488 as the detection probe (Figure 1). We also replaced glycerol with a DAPI-containing mounting medium, which allowed assessment of % fluorescence-labeled cells and correct interpretation of immunostaining patterns (Figure 6).

**Table 2:** A summary of the anti-NMDAR autoAb tests done with our optimized approach for patients suspected of anti-NMDAR-related autoimmune encephalopathy.

**Blood anti-NMDAR test results**	Number of blood samples tested^*^	Number of CSF samples tested^*^	**Number of CSF results matched to blood results^#^**	**%CSF-blood result matches**	**Number of double-stain tests performed**
Positive^**^	**37**	**20**	**17**	**85% (17/20)**	**5**
*initial titer 1:10*	*17*	*9*	*8*	89% (8/9)	*1*
*initial titer 1:32*	*13*	*4*	*3*	75% (3/4)	*1*
*initial titer 1:100*	*4*	*4*	*3*	75% (3/4)	*1*
*initial titer 1:320*	*3*	*3*	*3*	100% (3/3)	*2*
**Negative**	**39**	**8**	**8**	**100% (8/8)**	**2**

* *All patients suspected of anti-NMDAR encephalitis or referred by other hospitals were first tested with blood samples using our modified protocol based on EUROIMMUN IIFT (as outlined in Figure 7). Suspected patients presenting milder symptoms (e.g., predominantly psychiatric presentation) did not usually provide CSF samples, unless their blood test results later suggested a likelihood for the disease. So the number of CSF testing was lower than that of blood testing for the negative and lower titer groups*.

** *The initial titer was generally determined with the blood sample retrieved when a patient was first suspected of anti-NMDAR encephalitis or referred by other hospitals. We only provide positive or negative findings for CSF samples*.

# *The three cases that show discordance between blood and CSF test results were all due to negative CSF findings but positive blood findings*.

Indeterminate results (Figure 7) may cast doubts on whether some component in the clinical sample could indeed bind specifically to NMDA receptor. So if a significant fraction of the control cells that lack NMDAR expression (e.g., >5%) is labeled with green fluorescence, this is NMDAR-independent binding and could confound interpretation of the results from NMDAR+ cells (Tester BIOCHIPs). If the fraction of fluorescent cells exceeds 50% on the Tester BIOCHIP, this result should also be dealt with caution since the fraction of NMDAR+ cells on a commercial Tester BIOCHIP rarely exceeds 50% (Figure 7). Additionally, if the fluorescent pattern is atypical that of the normal expression of a surface receptor (e.g., lack of plasma membrane expression), the fluorescent signal is likely NMDAR-independent, too.

Our workflow is supplemented with a “double-labeling” option in case when single-labeling experiments show “indeterminate” results and require verification. Double labeling with the well-characterized mouse anti-NMDAR mAb marks the subcellular location of heterologously-expressed NMDA receptor, allowing an experimenter to determine whether the fluorescent signal is NMDAR-specific or not by direct visual assessment of the merged image of green and red fluorescence (Figure 7). As compiled in Table 2, the double-labeling tests were performed either for validation of ambiguous results from standard single stain, or for assessment of possible epitope shifts, particularly in serious, recurrent patients.

## Discussion

The recent emergence of anti-NMDAR encephalitis (28), which is frequently encountered in psychiatric services, reminds us the challenges in differential diagnosis of schizophrenia spectrum disorders. The similar clinical presentations between schizophrenia and the early phase of anti-NMDAR encephalitis also raise questions on whether their pathobiological mechanisms could overlap to a certain degree. But whether anti-NMDAR autoAbs are significantly present in patients with acute psychosis or schizophrenia remains highly controversial, in part due to complexities of patient samples and mediocre sensitivity/specificity of the current mainstream method for anti-NMDAR diagnostic bioassay. Here, we tackled the latter technical issue, and developed approaches to increase its sensitivity and accuracy.

We experimentally showed that replacement of secondary probe FITC with superior Alexa fluor 488 enhanced detection sensitivity (Figure 1). From our experience, we also recommend the use of an advanced fluorescence imaging system, which generally provides higher image resolution and sensitivity than a basic fluorescence microscope (Figure 2).

To improve the accuracy of anti-NMDAR diagnostics, we developed two validation protocols for samples with initial ambiguous results. Both protocols employed second labeling with a mouse anti-NR1 mAb, after clinical sample labeling (Figure 3). The ensuing colocalization test helped validate or disprove uncertain results from the conventional single-stain method. These two verification protocols not only helped identify cases with “false-positive” results, but also allowed us to track whether the binding affinities of autoAbs from different stages of the disease had changed relative to the same mouse mAb (Figure 4 5). Simultaneous labeling with DAPI specified locations of cell nuclei and helped validate or differentiate autoimmune targets at the subcellular level (Figure 6). Last, these improvements on the diagnostic sensitivity and accuracy should reduce the extra efforts and cost required for repeated testing, especially for samples with indeterminate results from single stain.

Our workflow (Figure 7) provides a general guideline for cell-based detection of neuronal autoAbs. Because NMDA receptor is a large membrane protein, ideally the protein retains its native, membrane-bound conformation best when heterologously expressed in an appropriate mammalian cell line. Non-cell-based, conventional ELISA that requires purified NMDA receptor or its protein fragment or peptide as the source of antigen conceivably is not ideal for this purpose. Another emerging technology—Meso Scale Discovery Electrochemiluminescence (MSD-ECL), has the potential to detect multiple antibodies with ultra-sensitivity. For detection of neuronal autoAbs, MSD-ECL also needs to adopt cell-based expression systems for correctly-folded membrane proteins (autoantigens), similar to the widely-used BIOCHIP methodology. Despite all the technical difficulties ahead, multiplex MSD-ECL is perhaps the only approach that will allow simultaneous detection and differentiation of various types of autoAb-mediated autoimmune encephalitis that is much in need clinically.

## Limitations

In this method paper, we implemented modification to the mainstream anti-NMDAR autoAb diagnostic method to improve detection sensitivity and accuracy. This was a study aiming to optimize the current lab diagnostic protocols using clinical samples primarily from patients suspected of anti-NMDAR encephalitis referred by neurologists and psychiatrists. We thus did not screen the prevalence of anti-NMDAR autoAbs in a large cohort of psychiatric illness (such as schizophrenia).

Test for the presence of anti-NMDAR autoAbs is critical for the diagnosis of anti-NMDAR autoimmune encephalitis. However, the presence of anti-NMDAR autoAbs in one's body fluid does not equate to anti-NMDAR-mediated disease, if relevant clinical symptoms are lacking. This is because some autoAbs may not be pathogenic if they never encounter the antigen, or if their interaction with endogenous NMDA receptor does not affect normal functions of the receptor or have any pathophysiological impacts. AutoAbs can also be transient and exhibit epitope shifts. So when a test outcome is unexpected, because of the complexity of the disease or its lab diagnostics, re-test or double-labeling verification should be considered (Figure 7).

## Author contributions

KH designed the experiments and wrote the paper. N-CC, Y-JL, R-FT, and C-CC conducted the human trial. H-JL and Y-SL performed experiments and analyzed the data. KH and CC supported experimentation. SD and KH performed imaging analyses.

### Conflict of interest statement

The authors declare that the research was conducted in the absence of any commercial or financial relationships that could be construed as a potential conflict of interest.
