# Supplementary material for: Optimization of an Anti-NMDA Receptor Autoantibody Diagnostic Bioassay
Source: Front Neurol. 2018 Aug 22;9:661. doi: 10.3389/fneur.2018.00661 (PMC6113861; doi:10.3389/fneur.2018.00661)
Supplement: Supplementary file 1 [file Data_Sheet_1.pdf]

Supplemental Figure S1.

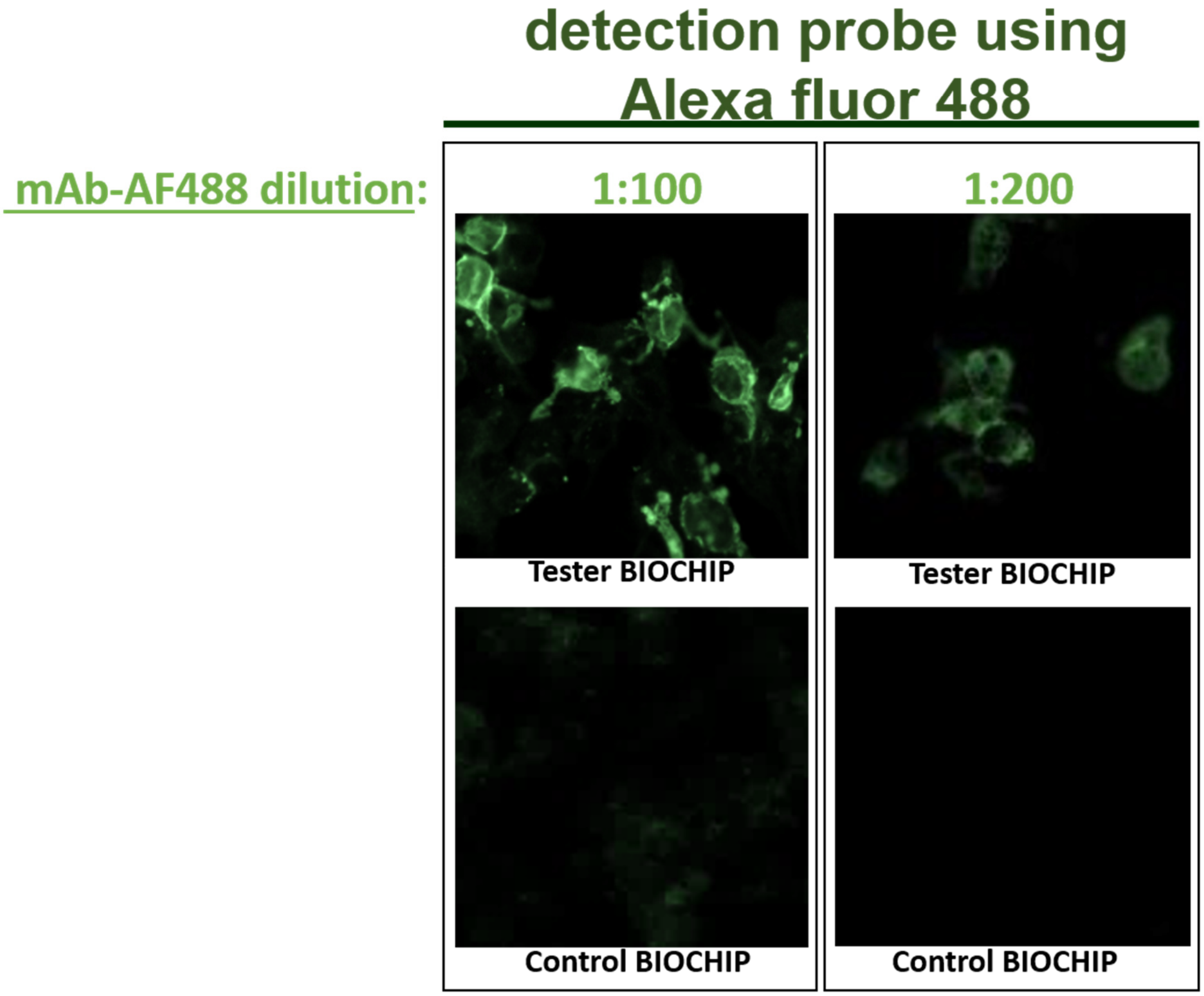

Figure S1. The most appropriate dosage of secondary anti-human mAb conjugated Alexa Fluor 488 (from Jackson ImmunoResearch Laboratories, Inc.) for use in conjunction with the BIOCHIPS from EUROIMMUN's Anti-Glutamate Receptor IIFT kit was determined to be at 1:100 dilution. A plasma sample from a patient with definite diagnosis of anti-NMDAR encephalitis was diluted 10-fold and incubated with two separate sets of BIOCHIPs (each set includes a TESTER BIOCHIP that was coated with NMDAR-expressing cells and a CONTROL BIOCHIP that was coated with negative cells). After one hour of incubation followed by PBST washes, one set was incubated with Alexa fluor 488-conjugated anti-human mAb at 1:100 dilution (left panel), and the other set with the same mAb probe at 1:200 dilution (right panel) for 30 minutes. Fluorescence imaging revealed an optimal secondary labeling at 1:100

dilution, at which unspecific binding of the sample to negative cells on the Control BIOCHIP remained minuscule.

Supplemental Figure S2.

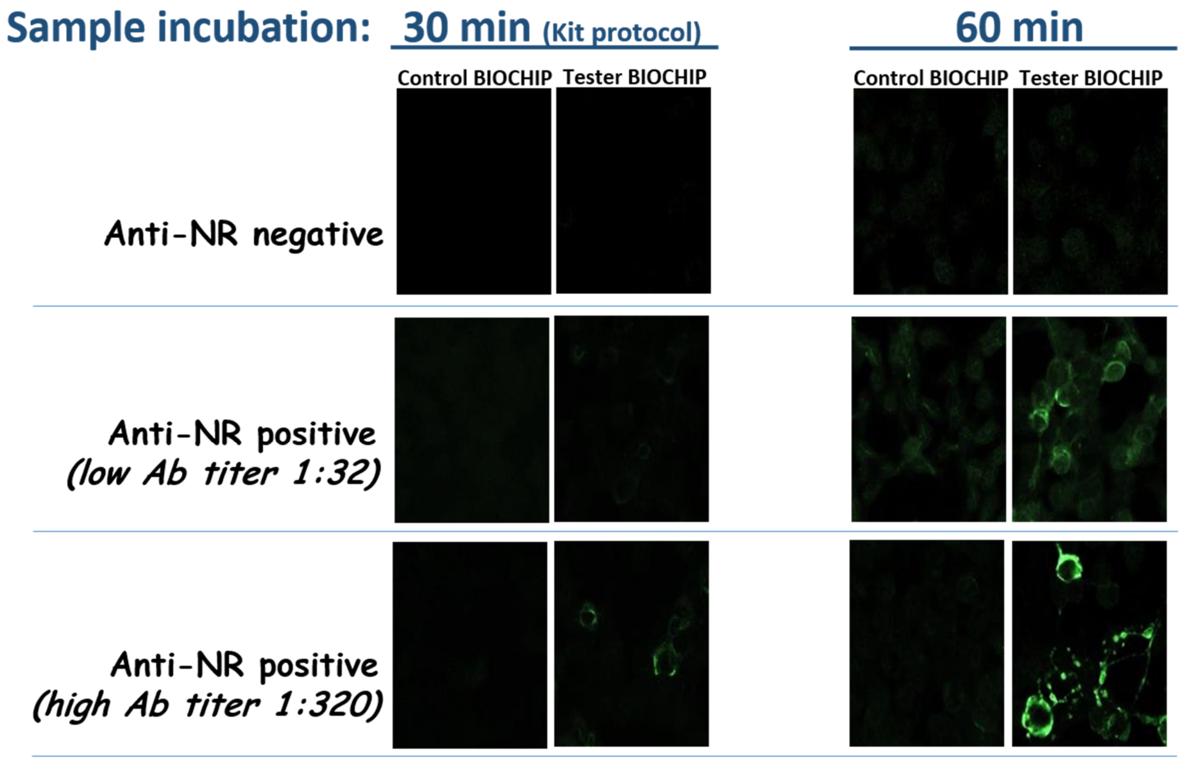

Figure S2. Extension of primary incubation time to 1 hour could help identify clinical samples with low antibody titers when used in conjunction with the EUROIMMUN's Anti-Glutamate Receptor IIFT kit. The kit protocol suggests 30 minutes for sample-BIOCHIP incubation. By doubling the incubation time to 1 hour, anti-NMDAR autoAbs at a low titer (such as that with titer 1:32 in the middle panel) could be clearly identified. Comparing to staining using an anti-NR-negative sample (top panel) or staining with the negative-control BIOCHIPs, extension of the sample incubation to 1 hour did not result in substantial non-specific binding. Secondary labeling here used anti-human mAb conjugated with Alexa fluor 488 (Jackson ImmunoResearch Laboratories, Inc.), and optimization of the secondary labeling was shown in Figure S1.

Supplemental Figure S3.

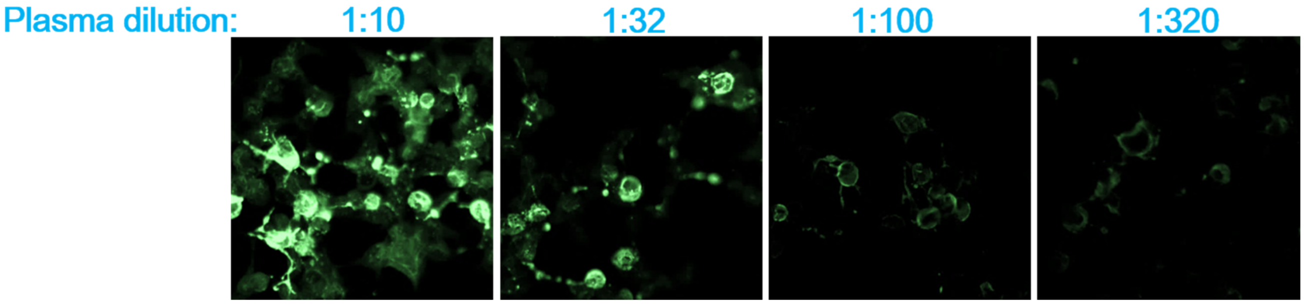

Figure S3. Anti-NMDAR diagnostics using a blood sample generally starts at 1:10 dilution, as suggested by the EUROIMMUN kit manual. Using the plasma sample from a patient with most severe anti-NMDAR encephalitis, above images showed the results of the sample dilution at 1:10, 1:32, 1:100, and 1:320. No fluorescence was observed at 1:640 dilution, so the anti-NMDAR titer for this plasma sample was determined to be 1:320.

Supplemental Figure S4.

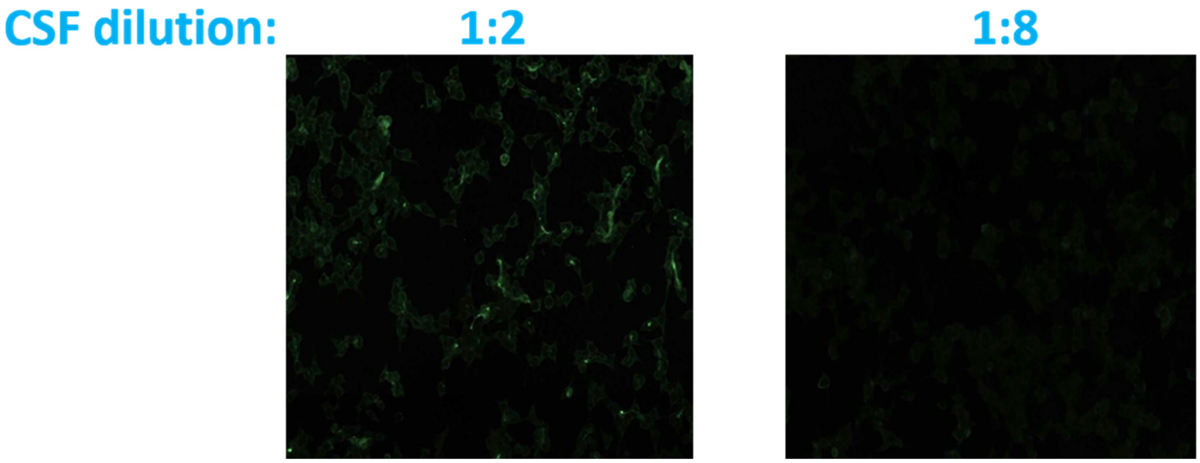

Figure S4. Anti-NMDAR diagnostics using a CSF sample generally starts at 1:2 dilution or with no dilution, as suggested by the EUROIMMUN kit manual. This CSF sample is from a patient with definite diagnosis of mild anti-NMDAR encephalitis, who was eventually cured by first-line immunosuppressive treatments. For this CSF sample, even though we used a more sensitive secondary probe Alexa fluor 488 and extended primary sample incubation from 30 minutes to 1 hour (Figures S1-S2), no fluorescence signaling could be found with further dilution of the CSF sample (1:8 dilution as shown).
